# Machine learning-based ability to classify psychosis and early stages of disease through parenting and attachment-related variables is associated with social cognition

**DOI:** 10.1186/s40359-021-00552-3

**Published:** 2021-03-23

**Authors:** Linda A. Antonucci, Alessandra Raio, Giulio Pergola, Barbara Gelao, Marco Papalino, Antonio Rampino, Ileana Andriola, Giuseppe Blasi, Alessandro Bertolino

**Affiliations:** 1 Department of Education, Psychology, Communication, University of Bari Aldo Moro, Via Scipione Crisanzio 42, 70122 Bari, Italy; 2 Department of Basic Medical Science, Neuroscience and Sense Organs, University of Bari Aldo Moro, Bari, Italy; 3 Lieber Institute for Brain Development, Johns Hopkins Medical Campus, Baltimore, MD USA; 4 Psychiatry Unit, Bari University Hospital, Bari, Italy

**Keywords:** Parental care, Parental overprotection, Adult attachment style, Machine learning, Schizophrenia, Bipolar disorder, Risk for psychosis

## Abstract

**Background:**

Recent views posited that negative parenting and attachment insecurity can be considered as general environmental factors of vulnerability for psychosis, specifically for individuals diagnosed with psychosis (PSY). Furthermore, evidence highlighted a tight relationship between attachment style and social cognition abilities, a key PSY behavioral phenotype. The aim of this study is to generate a machine learning algorithm based on the perceived quality of parenting and attachment style-related features to discriminate between PSY and healthy controls (HC) and to investigate its ability to track PSY early stages and risk conditions, as well as its association with social cognition performance.

**Methods:**

Perceived maternal and paternal parenting, as well as attachment anxiety and avoidance scores, were trained to separate 71 HC from 34 PSY (20 individuals diagnosed with schizophrenia + 14 diagnosed with bipolar disorder with psychotic manifestations) using support vector classification and repeated nested cross-validation. We then validated this model on independent datasets including individuals at the early stages of disease (ESD, i.e. first episode of psychosis or depression, or at-risk mental state for psychosis) and with familial high risk for PSY (FHR, i.e. having a first-degree relative suffering from psychosis). Then, we performed factorial analyses to test the group x classification rate interaction on emotion perception, social inference and managing of emotions abilities.

**Results:**

The perceived parenting and attachment-based machine learning model discriminated PSY from HC with a Balanced Accuracy (BAC) of 72.2%. Slightly lower classification performance was measured in the ESD sample (HC-ESD BAC = 63.5%), while the model could not discriminate between FHR and HC (BAC = 44.2%). We observed a significant group x classification interaction in PSY and HC from the discovery sample on emotion perception and on the ability to manage emotions (both *p* = 0.02). The interaction on managing of emotion abilities was replicated in the ESD and HC validation sample (*p* = 0.03).

**Conclusion:**

Our results suggest that parenting and attachment-related variables bear significant classification power when applied to both PSY and its early stages and are associated with variability in emotion processing. These variables could therefore be useful in psychosis early recognition programs aimed at softening the psychosis-associated disability.

**Supplementary Information:**

The online version contains supplementary material available at 10.1186/s40359-021-00552-3.

## Background

Schizophrenia and bipolar disorder with psychotic manifestations are two devastating psychosis-spectrum disorders that dramatically affect individuals’ quality of life and personal functioning [1]. Indeed, they are strongly associated with lifetime disability [2], reduced life expectancy [3], and increased relative risk of suicide [4]. Furthermore, they are both characterized by strong and largely overlapping anomalies in neurocognition and social cognition [5, 6], as well as by thought disturbances [7]. Given this evidence and the strong and long-lasting disability affecting individuals with psychosis (PSY), especially individuals suffering from schizophrenia or from bipolar disorder with psychotic manifestations, the identification of markers of disease to be targeted in early identification or prevention strategies aimed at softening the PSY-associated burden is becoming more and more crucial in the clinical psychology and personalized medicine fields.

In the pathophysiology of psychosis, environmental factors play a crucial role [8]. Amongst those, early adverse experiences that occurred in childhood, especially those related to psychological or physical abuse, are highly prevalent in individuals diagnosed with psychosis (PSY) [9]. Notably, childhood traumatic experiences are associated with more severe clinical profiles and higher functional impairments already in the early stages and risk conditions of mental illness [10]. According to previous literature, adverse childhood experiences are strongly associated with perceived negative parenting, which is in turn associated with attachment insecurity [11, 12]. Specifically, according to attachment theory [13], an innate motivational system prompts individuals to proximity seeking to alleviate distress. The quality of these experiences of proximity with significant others leads to the generation of individual cognitive-affective representations (i.e. “internal working models”) of self and others [14]. These models are key to affect regulation throughout the lifespan, as they guide how information from the social world is appraised, thus in turn potentially affecting social cognition abilities [15]. In adulthood, a secure attachment style, often originating from positive parenting experiences [12], is indeed associated with high distress tolerance and positive affect regulation [16]. On the other hand, interacting with unpredictable or insensitive attachment figures may make the development of a stable and secure mental foundation more difficult [17–19] and lead to attachment insecurity, declined either through attachment anxiety (e.g., individuals are strongly vigilant to social threats and rejection experiences, and tend to overestimate the impact of negative emotions), or through attachment avoidance (e.g., individuals disavow the need of being comforted by others, avoid closeness and intimacy, tend to suppress the impact of emotions) [20]. Given its association with affect regulation, it is not surprising that studies have consistently reported a significant association between attachment insecurity and social cognition impairments, a key characteristic of PSY [21, 22]. Furthermore, both negative parenting and attachment insecurity have been associated with reduced coping strategies and increased likelihood of emotional breakdowns [17]. In this framework, low parenting abilities, as well as attachment insecurity, have been previously considered as general environmental factors of vulnerability for mental illness [16] whose effects are amplified by other genetic and/or environmental factors [8, 23] within the psychosis risk pathways.

This evidence, therefore, suggests that both perceived parenting and attachment style may have value as potential targets of psychosis in early recognition strategies. Indeed, several univariate studies have reported significant associations between attachment insecurity and clinically relevant aspects of psychosis, e.g. negative symptoms [24], paranoia [25], negative beliefs [26–28], and social withdrawal [29]. However, univariate studies have characterized main effects or interactions between attachment security and psychosis only at the group-level, thus lacking in generalizability potential and underestimating inter-individual heterogeneity [30, 31]. Furthermore, little is known about the association of perceived negative parenting and attachment insecurity with earlier stages of psychosis, as well as with its risk conditions (i.e., clinical, familial). Thus, the extent of potential of parenting and attachment factors to be targeted in early intervention programs is still unclear. A strategy to circumvent these shortcomings is to employ machine learning techniques. Indeed, machine learning allows quantifying sensitivity, specificity and generalizability of a given set of variables at the single-subject level [32, 33], rather than just characterizing group differences. Therefore, employing machine learning to deeply investigate the discriminative power of parenting and attachment-related variables in both psychosis and early stages of disease would lead to a better understanding of how negative parenting and attachment insecurity might be considered core vulnerability factors of psychosis and its early stages. From a clinical perspective, this better understanding would potentially allow better tailoring of environmental factors within early identification programs. Specifically, accurate and generalizable machine learning models could potentially lead to the implementation of refined and individualized preventative interventions, and/or mental health promotion programs, which would in turn have a greater impact in the reduction of the burden associated with psychosis in terms of symptoms, quality of life, management [30].

Thus, the aim of this study is threefold: (i) to generate a parenting and attachment-based machine learning model which correctly discriminates between PSY and healthy controls (HC); (ii) to test whether this model could track the early stages of psychosis and/or risk conditions in independent samples; (iii) to investigate in both psychosis and its early stages the potential association between model’s performance and social cognition impairments. We hypothesized that both perceived parenting and attachment-related variables will bear significant PSY vs. HC classification performance. Furthermore, we hypothesized that this model will generalize to early stages of psychosis and familial risk conditions. Moreover, we expected that the discriminative ability of the model will be associated with social cognition abilities.

## Methods

### Sample determination

A total of 234 individuals, all Caucasians native of the Apulia region, Italy, participated in the study. Inclusion and exclusion criteria are reported in Supplementary Information, section "Background". The discovery sample was composed of 105 individuals, of which 71 HC and 34 PSY on stable antipsychotic treatment for at least one month (Table 1A). Of the PSY, 20 were diagnosed with schizophrenia and 14 with bipolar disorder with psychotic symptoms according to the Structural Clinical Interview for DSM IV-TR [34]. Moreover, 90 individuals were included in the validation clinical sample (Table 1B). Of those, 60 were HC and 30 were individual at the early stages of disease (ESD) compared with PSY. Specifically, 9 were labeled as First Episode of Depression, 9 as First Episode of Psychosis, and 12 as At-Risk Mental State for psychosis. A detailed description of the clinical characteristics of the ESD group is reported in Supplementary Information, section "Background". Furthermore, 26 HC and 13 individuals with a Familial High Risk for psychosis (FHR, i.e., with no DSM IV Axis I diagnosis, but with a first-degree relative affected either by schizophrenia or by bipolar disorder—Table 1C) were included in the validation familial risk sample. Both familial risk and clinical validation samples were used for replication purposes (section "Out-of-sample validation analyses"). ANOVA and χ^2^ were used to test for group differences in terms of demographics both within- and between- samples (Table 1).

**Table 1:** Demographic, clinical and neuropsychological characteristics of: (A) discovery sample; (B) validation clinical sample; (C) validation familial risk sampl

	All subjects (mean ± SD)	HC (mean ± SD)	PSY (mean ± SD)	HC vs. PSY p value
**A. Discovery sample**				
N	105	71	34	n.a
Gender Ratio (M/F)	58/47	35/36	23/11	0.119
Age	31.96 ± 10.14	28.82 ± 6.43	38.53 ± 13.05	< 0.001*
Socio-economic Status	40.58 ± 17.01	40.77 ± 17.61	40.18 ± 15.94	0.867
WAIS IQ	105.77 ± 15.97	111.04 ± 10.93	94.76 ± 19.13	< 0.001*
Premorbid IQ	114.27 ± 5.62	116.40 ± 2.69	109.80 ± 7.33	< 0.001*
GAF total score	51.79 ± 15.72	n.a	51.79 ± 15.72	n.a
Chlorpromazine eq	174.60 ± 110.13	n.a	174.60 ± 110.13	n.a

	All subjects (mean ± SD)	HC (mean ± SD)	ESD (mean ± SD)	HC vs. ESD p value
**B. Validation clinical sample**				
N	90	60	30	n.a
Gender ratio (M/F)	35/55	17/43	18/12	0.007*
Age	23.04 ± 6.33	22.95 ± 6.27	23.23 ± 6.56	0.843
Socio-Economic Status	42.62 ± 14.50	43.81 ± 14.78	40.25 ± 13.87	0.275
WAIS IQ	105.41 ± 11.26	106.60 ± 10.44	103.03 ± 12.61	0.158
Premorbid IQ	114.28 ± 3.55	115.33 ± 2.49	112.19 ± 4.39	0.300
GAF total score	64.04 ± 15.03	n.a	64.22 ± 15.34	n.a
Chlorpromazine eq	91.67 ± 46.87	n.a	95.45 ± 47.19	n.a

	All subjects (mean ± SD)	HC (mean ± SD)	FHR (mean ± SD)	HC vs. FHR *p* value
**C. Validation familial risk sample**				
N	39	26	13	n.a
Gender Ratio (M/F)	15/24	10/16	5/8	0.727
Age	25.41 ± 7.94	24 ± 5.12	28.23 ± 11.49	0.226
Socio-Economic Status	40.68 ± 13.50	38.19 ± 14.13	45.65 ± 10.99	0.105
WAIS IQ	108.13 ± 12.73	107.50 ± 13.71	109.38 ± 10.90	0.669
Premorbid IQ	115.70 ± 3.29	114.82 ± 3.01	117.46 ± 3.22	0.016*

Table 1D reports statistical comparisons between groups. Significant between-groups differences (*p* < 0.05) are marked with (*)

SD: Standard Deviation; HC: Healthy Controls; PSY = Patients with Psychosis; ESD = Early Stages of Disease; FHR = familial high-risk individuals M/F: Male/Female; IQ: Intelligence Quotient; PANSS: Positive and Negative Symptoms Scale; YMRS: Young Mania Rating Scale; n.a. = not assessed

### Perceived parental bonding and adult attachment assessment

All participants completed the Parental Bonding Instrument (PBI) for the assessment of the perceived parental bonding [35] (Supplementary Information, section "Methods"). The PBI is a 25-items self-report questionnaire investigating two main dimensions, “care” and “overprotection”, separately for maternal bonding and paternal bonding. The “care” dimension reflects perceived parental warmth, affection, and involvement in contrast to coldness, indifference, and rejection. On the other hand, the “overprotection” dimension reflects perceived parental psychological control and intrusion in contrast to the encouragement of autonomy and independence. The Italian version of the PBI shows good psychometric properties [36]. In this study, we focused on PBI continuous scores of separate maternal and paternal care and overprotection (4 variables in total) for analysis purposes.

To assess the adult attachment style, all individuals underwent the 36-items self-report questionnaire Experiences in Close Relationships Scale (ECR) [37]. The ECR allows researchers to investigate feelings and behaviors related to significant relationships in adulthood along two dimensions: "anxiety about abandonment" and "avoidance of closeness". The anxiety factor includes intense concerns for romantic relationships, fear of being abandoned and frequent requests to greater involvement of partners; the second factor, avoidance, includes difficult and uncomfortable feelings in managing emotions and in relying on partners. The Italian version of the ECR shows good psychometric properties [38]. In this study, we focused on continuous scores of ECR anxiety and avoidance for analysis purposes.

Two-sample t-tests were employed to assess differences in discovery and validation samples for each of the PBI and ECR variables of interest (Table 2). All significant p values were < 0.05.

**Table 2:** Features entering the machine learning algorithms and their respective characterization in all study samples

	All subjects (mean ± SD)	HC (mean ± SD)	PSY (mean ± SD)	HC vs. PSY p value
**A. Discovery sample**				
PBI maternal care	25.14 ± 6.39	25.77 ± 5.81	23.82 ± 7.39	0.144
PBI maternal overprot	16.30 ± 7.54	15.21 ± 7.09	18.56 ± 8.03	0.033*
PBI paternal care	20.57 ± 7.79	20.28 ± 7.75	21.18 ± 7.94	0.583
PBI paternal overprot	14.97 ± 7.22	13.80 ± 7.06	17.35 ± 7.07	0.018*
ECR avoidance	46.59 ± 16.28	44.06 ± 16.64	51.88 ± 14.32	0.020*
ECR anxiety	65.61 ± 21.22	61.30 ± 20.11	74.62 ± 20.92	0.002*

	All subjects (mean ± SD)	HC (mean ± SD)	ESD (mean ± SD)	HC vs. ESD p value
**B. Validation clinical sample**				
PBI maternal care	27.18 ± 6.49	28.67 ± 5.30	24.20 ± 7.63	0.002*
PBI maternal overprot	16.18 ± 6.65	15.32 ± 6.61	17.90 ± 6.49	0.082
PBI paternal care	22.76 ± 8.06	23.42 ± 7.68	21.43 ± 8.74	0.273
PBI paternal overprot	13.30 ± 7.09	13.35 ± 7.14	13.20 ± 7.11	0.925
ECR avoidance	44.37 ± 18.26	41.40 ± 17.59	50.30 ± 18.43	0.028*
ECR anxiety	66.17 ± 19.85	63.43 ± 19.58	71.63 ± 19.56	0.064

	All subjects (mean ± SD)	HC (mean ± SD)	FHR (mean ± SD)	HC vs. FHR p value
**C. Validation familial risk sample**				
PBI maternal care	26.33 ± 6.71	25.65 ± 6.88	27.69 ± 6.41	0.368
PBI maternal overprot	15.44 ± 7.62	17.27 ± 7.01	11.77 ± 7.71	0.032*
PBI paternal care	23.69 ± 6.53	24.50 ± 6.17	22.08 ± 7.18	0.280
PBI paternal overprot	14.28 ± 8.17	15.35 ± 7.46	12.15 ± 9.39	0.255
ECR avoidance	45.21 ± 16.42	43.88 ± 14.12	47.85 ± 20.67	0.542
ECR anxiety	61.51 ± 17.81	61.96 ± 16.63	60.62 ± 20.65	0.827

Significant between-groups differences (*p* < 0.05) are marked with (*)

PBI = Parental Bonding Instrument; ECR = Experiences in Close Relationships; overprot = overprotection; HC = Healthy Controls; PSY = Patients with Psychosis; ESD = Early Stages of Disease; FHR = Familial High Risk; SD = standard deviation

### Social cognition assessment

All individuals were administered three tests, each aimed at investigating a different aspect of socio-cognitive abilities:

- to investigate emotion perception, we employed the Facial Emotion Identification Test (FEIT) ([39]; details about the Italian version employed can be found at [40]); we employed a computerized FEIT version [41] in which individuals were shown 19 individuals' faces each depicting one of six different emotions (happiness, sadness, anger, surprise, fear, shame), shown one at a time for 15 s, with 10 s of blank screen between each stimulus presentation. 15 photographs depict negative emotions (sadness, anger, fear, and shame), while 4 photographs depict positive emotions (happiness, and surprise). After the presentation of each stimulus, individuals were required to select which of the six emotions was depicted on the picture and to mark it on an answer form. The total test score was computed as the percentage of correct answers.
- to investigate theory of mind, we employed The Awareness of Social Inference Test (TASIT) ([42]; details about the Italian version employed can be found at [40]). The TASIT is composed of seven scales (positive emotions, negative emotions, sincere, simple sarcasm, paradoxical sarcasm, sarcasm enriched, lie), organized into three sections: in the section “emotion recognition”, individuals undergo 28 video-vignettes of professional actors enacting ambiguous scripts representing 7 basic emotions (happy, sad, surprised, angry, anxious, revolted, neutral), and at the end of each vignette they are asked to choose the perceived emotion of a given actor indicated in the answer form. The second section, social inference (minimal), allows researchers to investigate the understanding of conversational meanings that are determined by paralinguistic cues. Here, individuals are required to watch 15 video-vignettes of either sincere or sarcastic or paradoxical everyday conversational exchanges; for each vignette, individuals are asked 4 comprehension questions, respectively testing the understanding of actors’ beliefs, meaning, intentions, and feelings. The last section, social inference (enriched), assesses the ability to use contextual knowledge, like visual and verbal information, to derive meaning. Individuals are asked to undergo 16 video-vignettes, each one including a literally untrue comment (see Supplementary Information, section "Results" for a full description). For each vignette, individuals are asked 4 comprehension questions, respectively testing the understanding of actors’ beliefs, meaning, intentions, and feelings. For analysis purposes, we used the total number of correctly answered questions for each of the three TASIT sections.
- To test the ability to manage emotions, i.e. “*to be open to feelings, and to modulate them in oneself and others so as to promote personal understanding and growth of regulating emotions in oneself and in one’s relationships with others*” [43], we employed the Branch 4 (managing emotions) of the Mayer–Salovey–Caruso Emotional Intelligence Test (MSCEIT) ([43], Italian version: [44]). MSCEIT Branch 4 allows researchers to examine the ability to manage emotions by asking individuals to read short stories about an imaginary person going through an emotionally difficult situation, and then determine how effective several different courses of action would be for that given person in coping with the difficult emotions of the story. Individuals rate every possible action ranging from «Very ineffective», to «Very effective». MSCEIT scoring was based on the consensus scoring methods outlined in the manual [43].

Two-sample t-tests were employed to assess differences in discovery and validation samples for FEIT, TASIT, and MSCEIT total scores employed in subsequent statistical analysis (Table 3). All p values were <0.05. In the case of significant results, TASIT p values were <0.05, False Discovery Rate (FDR) corrected for the number sections tested (i.e., 3) [45].

**Table 3:** Social Cognition performance in all study samples

	All subjects (mean ± SD)	HC (mean ± SD)	PSY (mean ± SD)	HC vs. PSY p value
**A. Discovery sample**				
FEIT %correct	77.82 ± 10.66	82.004 ± 7.83	69.09 ± 10.57	< 0.001*
TASIT tot.corr. Section 1	24.13 ± 3.22	25.34 ± 1.93	21.62 ± 3.97	< 0.001*
TASIT tot.corr. Section 2	48.37 ± 8.45	51.42 ± 6.40	41.79 ± 8.67	< 0.001*
TASIT tot.corr. Section 3	49.56 ± 7.11	53.04 ± 3.71	42.38 ± 7.07	< 0.001*
MSCEIT Br4 total score	83.04 ± 9.84	85.75 ± 9.47	77.39 ± 8.16	< 0.001*

	All subjects (mean ± SD)	HC (mean ± SD)	ESD (mean ± SD)	HC vs. ESD p value
**B. Validation clinical sample**				
FEIT %correct	79.27 ± 8.18	80.43 ± 7.21	76.96 ± 9.57	0.057
TASIT tot.corr. Section 1	25.12 ± 2.41	25.40 ± 2.33	24.57 ± 2.50	0.122
TASIT tot.corr. Section 2	50.85 ± 4.93	52.20 ± 4.17	48.17 ± 5.30	< 0.001*
TASIT tot.corr. Section 3	50.10 ± 5.01	51.25 ± 3.56	47.80 ± 6.58	0.011*
MSCEIT Br4 total score	86.49 ± 9.78	87.44 ± 10.01	84.58 ± 9.17	0.193

	All subjects (mean ± SD)	HC (mean ± SD)	FHR (mean ± SD)	HC vs. FHR p value
**C. Validation familial risk sample**				
FEIT %correct	80.02 ± 1.32	80.03 ± 1.36	80 ± 1.27	0.942
TASIT tot.corr. Section 1	23.83 ± 1.14	24 ± 1.30	23.50 ± 0.64	0.200
TASIT tot.corr. Section 2	51.28 ± 4.16	56.50 ± 1.85	48.67 ± 1.84	< 0.001*
TASIT tot.corr. Section 3	53.83 ± 2.01	54 ± 2.42	53.50 ± 0.64	0.471
MSCEIT Br4 total score	82.25 ± 5.08	87.41 ± 3.36	83.93 ± 7.04	0.043*

Significant between-groups differences (p < 0.05) are marked with (*)

PBI = Parental Bonding Instrument; FEIT = Facial Emotion Identification task; TASIT = The Awareness Of Social Inference Test; MSCEIT = Mayer–Salovey–Caruso Emotional Intelligence Test; HC = Healthy Controls; PSY = Patients with Psychosis; ESD = Early Stages of Disease; FHR = Familial High Risk; SD = standard deviation

### Machine learning pipeline

The overall machine learning strategy was carried out through the NeuroMiner software, version 1.0 (https://github.com/neurominer-git?tab=repositories) and consisted in building a multimodal algorithm based on the six perceived parental bonding and adult attachment related-variables (i.e., PBI maternal care, PBI paternal care, PBI maternal overprotection, PBI paternal overprotection, ECR avoidance, ECR anxiety, hereby called “features”, Table 2) which accurately discriminates between HC and PSY. With this aim, we implemented a repeated nested cross-validation strategy (CV, Supplementary Information, section "Discussion") [32, 46, 47] to identify models that contributed most to the classification pattern separating PSY and HC at the inner CV level. To enforce an unbiased estimation of classification generalizability, these models were then applied to the test data at the outer CV level, which included individuals that were not used for training the classification algorithm [48]. We obtained a Support Vector Machine [49] ensemble model based on the six perceived parental bonding and adult attachment-related variables listed before. Model performance was measured using sensitivity, specificity, balanced accuracy (BAC), positive predictive value (PPV), negative predictive value (NPV), and Area-Under-the Curve (AUC) based on the class membership probability scores generated through ensemble-based majority voting in the repeated nested CV framework (Supplementary Information, section Discussion). We also assigned statistical significance to the observed classification performance of our model through permutation analysis (Supplementary, Information, sect. 5). Furthermore, to understand the importance of the input features for generating decisions (i.e., PSY or HC?), we computed for each feature the probability of being selected for classification purposes within the inner CV loop [50, 51]. A detailed description of our machine learning pipeline is reported in Supplementary Information, section "Discussion".

Further, we performed some sanity checks in order to exclude that our machine learning algorithm was associated with any clinical confound. Therefore, in the PSY group, we conducted Pearson’s r correlations between subject-specific linear SVM decision scores and, respectively, medication (expressed in chlorpromazine equivalents), level of functioning (as assessed by Global Assessment of Functioning, [52]), and age of onset. A more positive decision score suggests that a given individual is highly prototypical of the HC class, while a more negative decision score suggests that a given individual is highly prototypical of the PSY class.

### Out-of-sample validation analyses

To investigate whether the machine learning model can discriminate between PSY and HC could have a potential prognostic validity, we externally validated the model in the validation clinical sample (Table 1B) and in the validation familial risk sample (Table 1C). With this purpose, we performed out-of-sample validation analysis by applying the SVM ensemble decision model obtained from the discovery analysis without any in-between re-training steps to both validation cohorts (i.e., ESD vs. HC, and FHR vs. HC), as already done in previous publications [51, 53].

### Association analysis between decision scores and social cognition

To investigate whether the classification performance of our algorithm was associated with social cognition performance as a function of diagnosis, we tested the diagnosis (i.e., PSY vs. HC) x classification rate (i.e., correctly classified vs. misclassified individuals, based on the comparison between observed and predicted labels) interaction on social cognition (as measured by FEIT total score, TASIT total score, MSCEIT standardized total score). Analyses were controlled for age, gender and premorbid IQ (Supplementary Information, section "Background"). All significant *p* values were *p* < 0.05. In the case of significant results, TASIT p values were < 0.05, FDR corrected for the number of sections tested (i.e., 3) [45].

Given the validation results (see Results, section "Validation familial risk sample"), this same analysis pipeline has been repeated on the validation clinical sample.

As a sanity check, to rule out any possibility that differences in terms of social cognition performance across groups (Table 3) could have influenced the classification performance of our algorithm, we performed correlation analysis on the whole discovery sample between all features which entered the machine learning algorithm, and each socio-cognitive variable of interest used for association analysis. All p values were > 0.05, FDR corrected. Results of this sanity check are reported in Supplementary Table S2 and highlight the absence of any significant association between features entering the algorithm and socio-cognitive variables of interest.

## Results

### Discovery sample

**Fig. 1:**
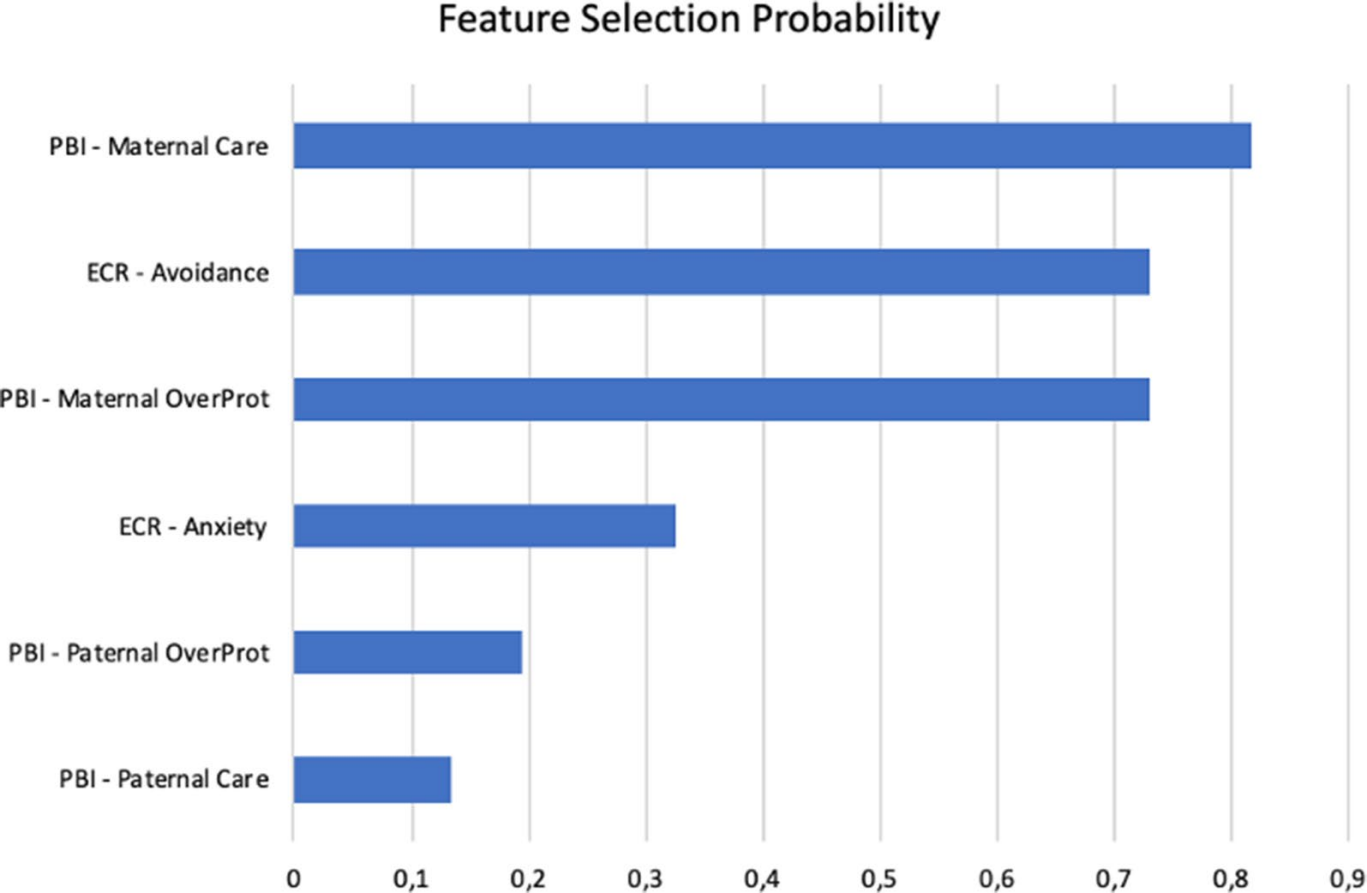
Probability of each feature for being selected in the machine learning Cross-Validation framework. Score closer to 1 represent a higher probability of being selected for decision by the Support Vector Machine algorithm. Abbreviations: PBI = Parental Bonding Instrument; ECR = Experiences in Close Relationships

ANOVA and χ^2^ analyses revealed that PSY differed significantly from HC in terms of age, WAIS and premorbid IQ (all *p* < 0.001, Table 1A), as well as on all measures of social cognition (all *p* < 0.001, Table 3A), with PSY performing significantly worse than HC. The machine learning model based on parental bonding and adult attachment-related variables correctly discriminated PSY from HC with a cross-validated BAC of 72.2% (sensitivity: 82.4%; specificity: 62.2%; Area Under the Curve: 0.71) and was significant at *p* < 0.001. Detailed statistics of all individual classification models are reported in Table 4. When computing the probability of each feature of being selected for classification purposes within the inner CV loop, we observed that the features with the highest probability (i.e., those that were selected the most for discrimination purposes within the CV framework) were PBI maternal care, ECR avoidance, and PBI overprotection (Fig. 1). In PSY, we found no association between SVM decision scores and, respectively, medication, GAF score and age of onset (respectively, *p* = 0.25, *p* = 0.52, *p* = 0.87).

**Table 4:** Validated classification performance of individual classifiers in all study cohorts

Stydy Cohort	True Negatives	True Positives	False Negatives	False Positives	Sensitivity	Specificity	Balanced Accuracy	Area Under the Curve	Positive Predictive Value	Negative Predictive Value	Positive Likelihood Ratio	Permutation Test, p value
Discovery Sample (HC vs. PSY)	44	28	6	27	82.4	62.0	72.2	0.71	50.9	88.0	3.5	< 0.001
Validation Clinical Sample (HC vs. ESD)	34	19	11	26	67.7	59.3	63.2	0.63	46.6	77.7	1.8	n.a
Validation Familial Risk Sample (HC vs. FHR)	9	7	6	17	53.8	34.6	44.2	0.46	29.1	60.0	0.8	n.a

HC = Healthy Controls; PSY = Patients with Psychosis; ESD = Early Stages of Disease; FHR = Familial High Risk

### Validation samples

#### Validation clinical sample

ANOVA and χ^2^ revealed that ESD differed significantly from HC in terms of gender distribution (*p* = 0.007, Table 1B) and of social inference performance (minimal, *p* < 0.001; enriched, *p* = 0.011) as measured by TASIT sections "Methods" and "Results" (Table 3B), with ESD performing worse than HC. Results from the out-of-sample validation analysis discriminating between ESD and HC based on the model classifying PSY from HC (Results section "Discovery sample") revealed that the parental bonding and adult attachment-related model discriminating between PSY and HC also discriminated between ESD and HC with a cross-validated BAC of 63.5% (sensitivity: 67.7%; specificity: 59.3%; Area Under the Curve: 0.65, Table 4).

#### Validation familial risk sample

ANOVA and χ^2^ revealed that FHR differed significantly from HC in terms of IQ (*p* = 0.016, Table 1C) and of minimal social inference (TASIT section "Methods", *p* < 0.001, Table 3C) and managing of emotions abilities (MSCEIT Branch 4, *p* = 0.043, Table 3C), with FHR performing worse than HC. Results from the out-of-sample validation analysis discriminating between FHR and HC based on the model classifying PSY from HC revealed that the parental bonding and adult attachment-related model discriminating between PSY and HC did not significantly discriminate between ESD and HC (cross-validated BAC: 44.2%, Table 4).

### Association analysis between decision scores and social cognition

#### Discovery sample

**Fig. 2:**
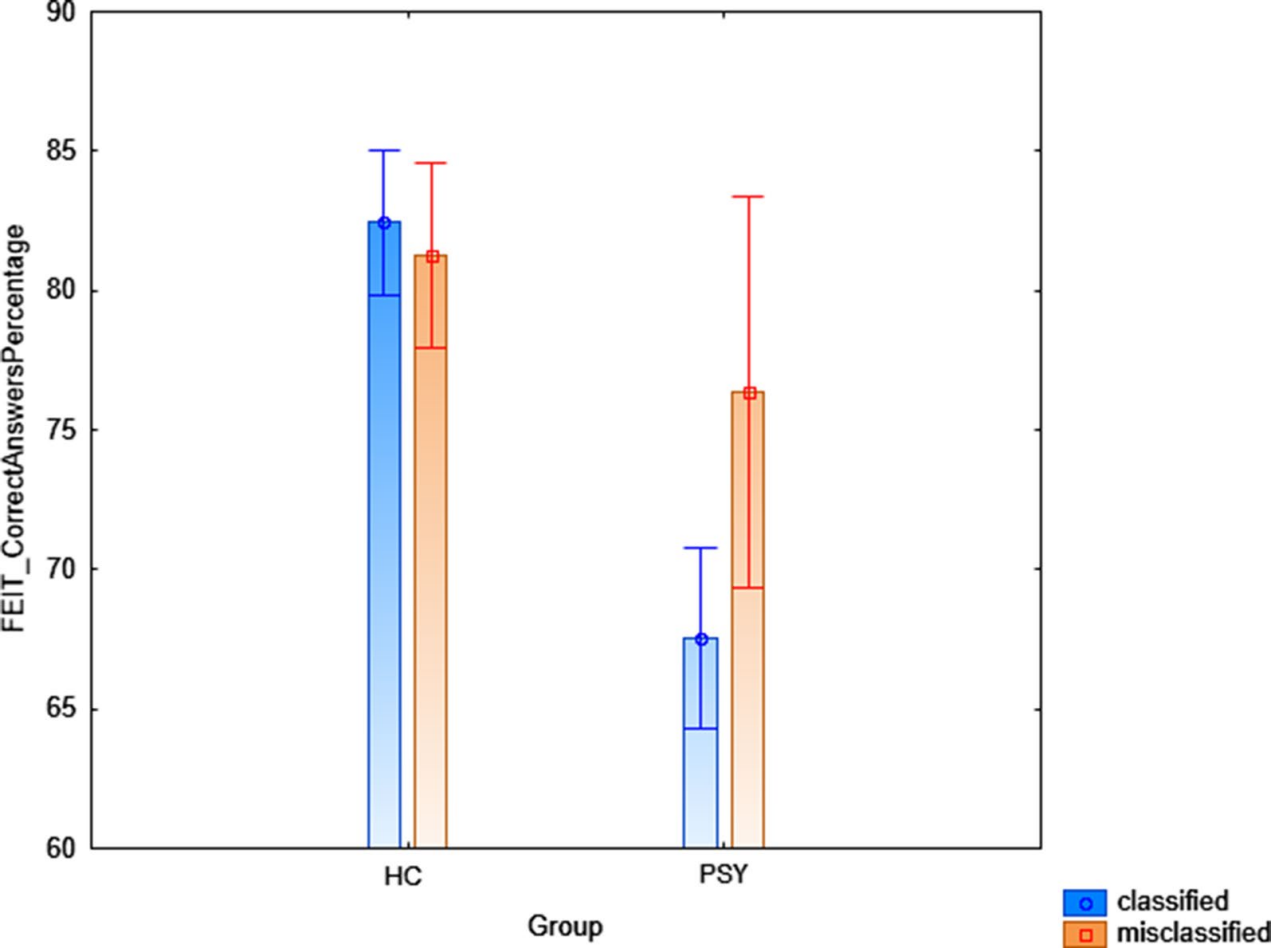
Depicting of the group x classification rate interaction on FEIT percentage of total correct responses in the discovery sample. Abbreviations: FEIT = Facial Emotion Identification Test; PSY = Individuals diagnosed with Psychosis; HC = Healthy Controls

**Fig. 3:**
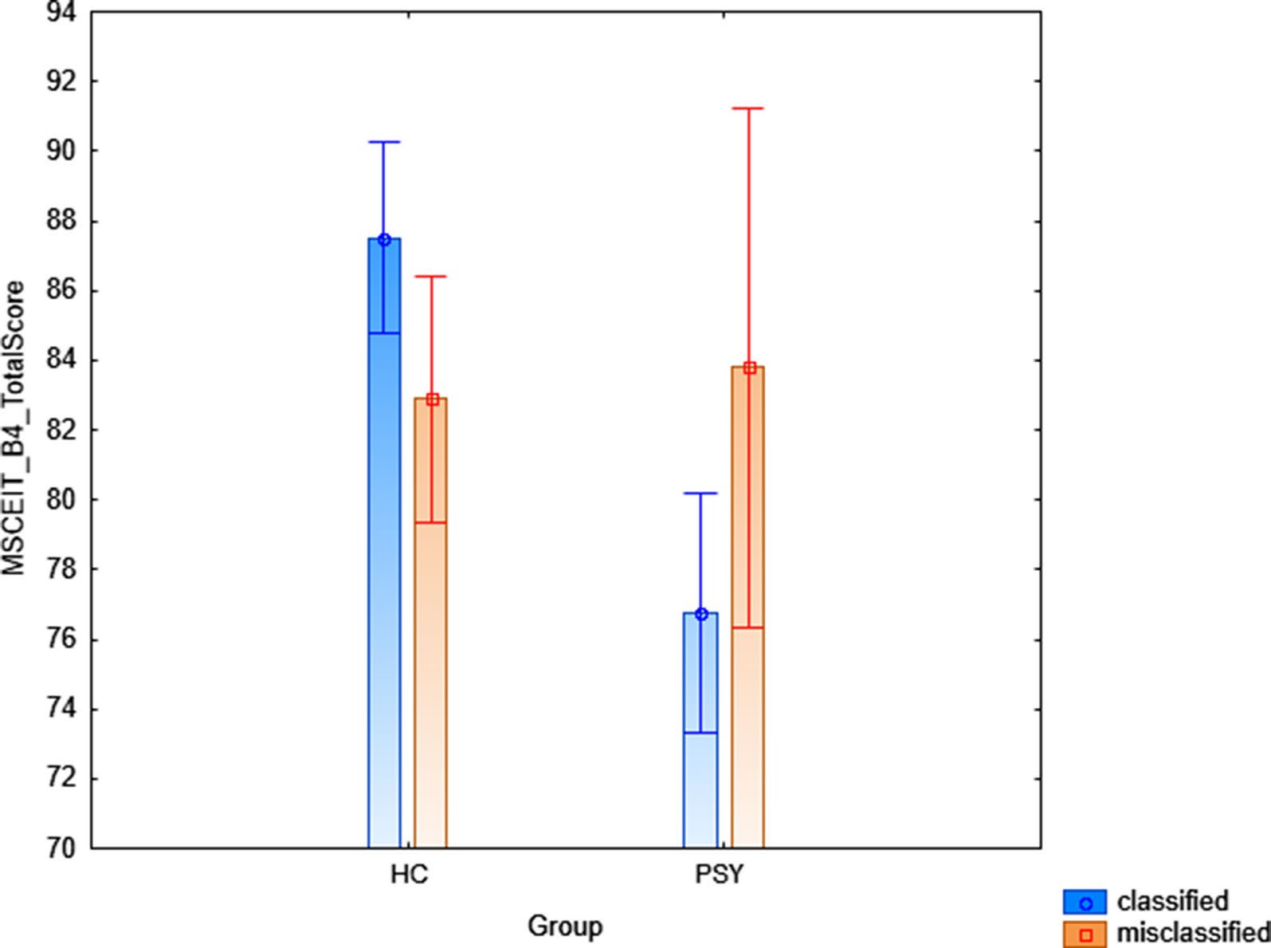
Depicting of the group x classification rate interaction on MSCEIT total score in the discovery sample. Abbreviations: FEIT = Mayer Salovey Caruso Emotional Intelligence Test; PSY = Individuals diagnosed with Psychosis; HC = Healthy Controls

General linear models revealed a significant diagnosis x classification rate interaction on FEIT percentage of correct answers (F = 5.11, *p* = 0.02, Fig. 2), such that the misclassified PSY (i.e., the PSY individuals that the algorithm wrongly identified as HC) had higher percentage of correct responses than the correctly classified PSY (i.e., the PSY individuals that the algorithm correctly identified as PSY) (*p* = 0.03), while no differences between the correctly classified and the misclassified HC individuals were found (*p* = 0.58). We also found a significant diagnosis x classification rate interaction on MSCEIT Branch 4 total score (F = 6.39, *p* = 0.02, Fig. 3), such that the misclassified HC (i.e., the HC individuals that the algorithm wrongly identified as PSY) had lower MSCEIT total score than the correctly classified HC (i.e., the HC individuals that the algorithm correctly identified as HC, *p* = 0.04). On the other hand, no differences between the correctly classified and the misclassified PSY individuals were found (*p* = 0.09). No significant interactions were found on TASIT section-related total scores (respectively, *p* = 0.45, *p* = 0.31, *p* = 0.15).

#### Validation clinical sample

**Fig. 4:**
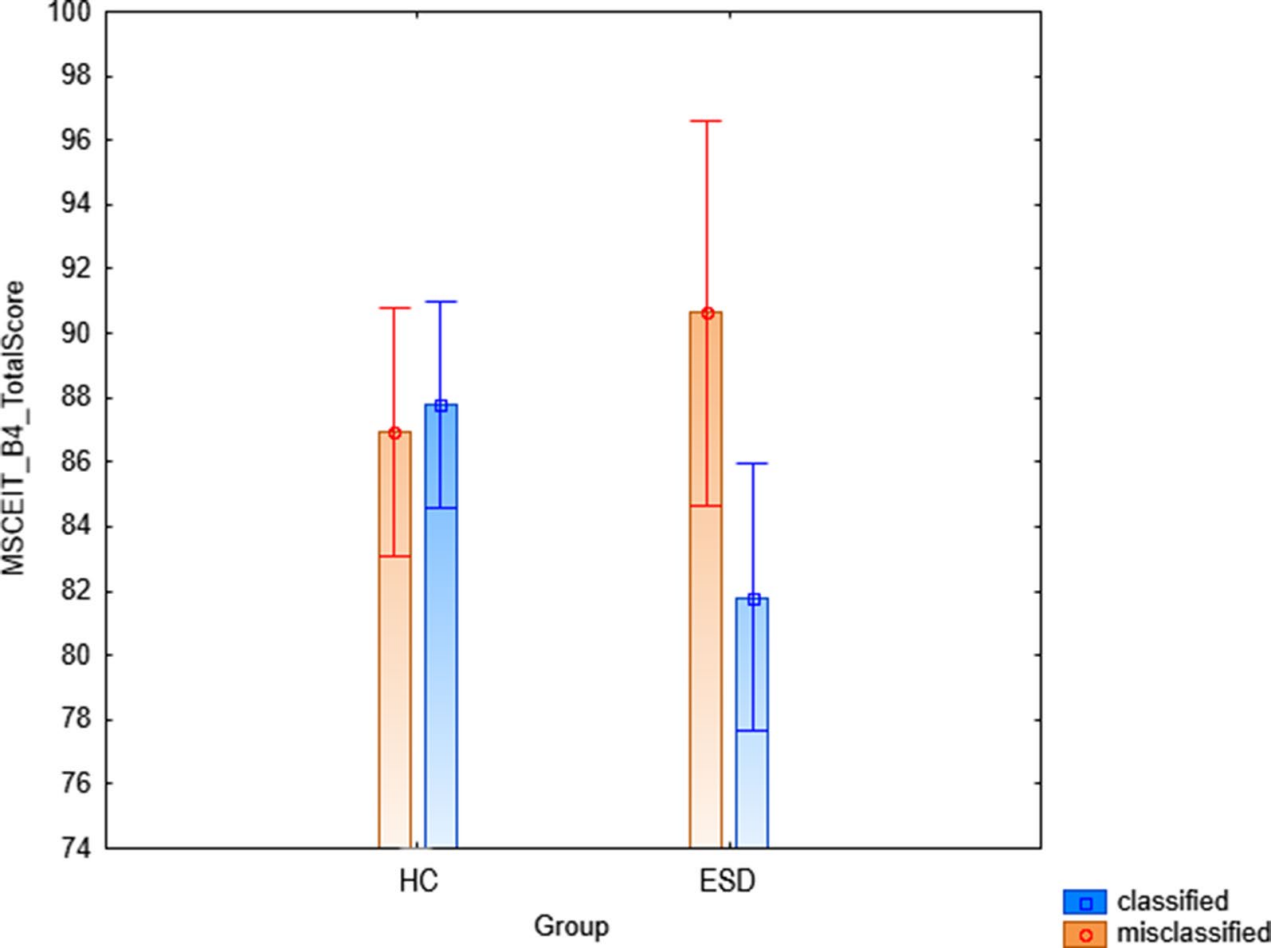
Depicting of the group x classification rate interaction on MSCEIT total score in the validation clinical sample. Abbreviations: FEIT = Mayer Salovey Caruso Emotional Intelligence Test; ESD = Early Stages of Disease; HC = Healthy Controls

General linear models revealed a significant diagnosis x classification rate interaction on MSCEIT Branch 4 total score (F = 4.74, *p* = 0.03, Fig. 4), such that the misclassified ESD (i.e., the ESD individuals that the algorithm wrongly identified as HC) had higher MSCEIT total score than the correctly classified ESD (i.e., the ESD individuals that the algorithm correctly identified as ESD) (*p* = 0.02), while no differences between the correctly classified and the misclassified HC individuals were found (*p* = 0.74). No significant interactions were found on FEIT percentage of correct responses (*p* = 0.40) and on TASIT section-related total scores (respectively, *p* = 0.06, *p* = 0.16, *p* = 0.51).

## Discussion

In this study, we aimed at discriminating between PSY and HC within a machine learning framework based on perceived parenting and attachment-related variables, at externally validating this classification model in cohorts reflecting early stages of psychosis (i.e., ESD individuals), as well as its familial risk (i.e., FHR individuals), and at understanding across these cohorts whether algorithmic decisions were associated with social cognition impairments. To the best of our knowledge, our results for the first time offer preliminary insights about the machine learning-based classification ability of perceived quality of parenting and attachment style variables, revealing that this set of variables correctly discriminates between PSY and HC with a 72.2% BAC and significance. These findings are in line with previous literature suggesting the role of a perceived negative family environment [54] and insecure attachment [16] in the vulnerability for psychosis. Notably, the most reliable features for classification purposes were PBI maternal care, ECR attachment avoidance and PBI maternal overprotection. The PBI results are in line with previous evidence showing that expressed emotions, communication deviance, and rearing within the family have all been previously associated with the prognosis of psychosis [54]. As concerns attachment avoidance, it has been previously associated with both positive and negative symptoms in PSY [24], and with psychotic symptoms, paranoia, endorsement of delusional experiences, negative affect regulation and social anhedonia in non-clinical samples [25, 55]. Nevertheless, these results should be taken with caution. Indeed, the risk pathways of psychosis are complex and heterogeneous, and reflect complex interplays between multiple genetic, environmental and neurocognitive characteristics [23]. In this framework, our results do not suggest that perceived negative parenting and attachment insecurities are sufficient causative factors for psychosis. Rather, they highlight their potential role as general vulnerability-to-psychosis factors[16], whose effect may be amplified by other more specific schizophrenia and bipolar disorder-related genetic and environmental factors, possibly leading to cognitive biases in reality interpretation [23].

This view is further corroborated by the fact that our perceived parenting and attachment-related model correctly discriminated also unseen ESD and HC individuals with 63.5% BAC. The 8.7% BAC drop observed in comparison with the discovery sample’s performance may be explained by both clinical reasons (i.e., the ESD cohort is composed by individuals either at their first episode of psychosis or depression or by individuals at high clinical risk for psychosis, see Supplementary Information, section "Background) and demographic differences between the discovery and the validation clinical cohorts (Table 1). Despite these differences, these findings show that our model not only achieves high diagnostic performance, but it also carries an intrinsic early identification potential. Thus, they may speak in favor of the possibility of specifically targeting parenting and attachment-related factors into early identification and prevention programs, especially in terms of promotion of attachment security as a key general resilience factor [56]. Nevertheless, future studies in larger and geographically diverse samples are warranted to realistically understand the potential of translation into clinical practice of our results.

It should be also noted that the model we generated in the PSY and HC discovery sample discriminated between FHR and HC at chance-level, thus suggesting that the general vulnerability-to-psychosis role of parenting and attachment characteristics might act only in the “late” portion of psychosis risk (as testified by the validation in ESD individuals), but not in “early” portions of psychosis risk. Indeed, FHR individuals do not have any clinical impairment or other risk conditions, but their PSY first-degree relative. This view is supported by previous evidence in which significant avoidance differences in attachment anxiety and avoidance have been found between individuals diagnosed with schizophrenia and their unaffected siblings, but not between their siblings and controls [22]. Another aspect that should be taken into consideration is that the PBI and ECR instruments, from which the features entering the machine learning algorithm were extracted, are self-report questionnaires. ﻿Despite evidence testifying that attachment self-reports measures are highly reliable in reflecting individuals’ actual attachment dispositions [57, 58], it can’t be excluded that the internal working models of attachment experiences, as well as the perception of the parenting received, are affected by biases in reality interpretation associated with illness experience at any stage (i.e., in both PSY and ESD individuals), which should not occur when only familial or genetic risk conditions are present. Indeed, a previous contribution [59] showed that individuals with an ongoing depressive disorder had more negative recollections of parenting experiences than those who remitted from a depressive disorder. This evidence corroborates the hypothesis that the experience of a mental illness is associated with biases in the interpretation of memories, although it is difficult to attribute any causality or directionality to this association. Nevertheless, our findings testify the urgency of implementing psychoeducation actions in psychosis, potentially through the combination of social skills trainings and emotional/motivational support to all family members, aimed at improving illness knowledge, resilience and coping skills after adverse childhood experiences. These actions would support not only individuals suffering from psychosis but would also more likely alleviate the illness-related emotional burden of their caregivers.

We also found a significant interaction between group and classification rate on social cognition performance in the cohorts in which our models showed good classification power, which speaks in favor of targeting social cognition deficits in early identification and intervention programs through social skills trainings. Indeed, in PSY and HC individuals from the discovery sample, this interaction was present on both FEIT and MSCEIT total scores. For FEIT, PSY that the algorithm misclassified as HC had higher emotion perception and ability to manage emotions, compared with PSY correctly classified as cases, and not as controls. For MSCEIT, HC individuals that the algorithm misclassified as PSY had lower emotion perception and managing of emotions abilities, compared with HC individuals that correctly classified as controls, and not as cases. We found the interaction also in the ESD and HC validation clinical sample, but only on MSCEIT total score, such that misclassified ESD had higher managing of emotions ability compared with correctly classified ESD. In both samples, no group x classification rate interaction was found on theory of mind abilities. Taken together, these results support the view for which parenting and attachment experiences guide how the social world is appraised [15] to the extent that their association with social cognition abilities is significant. Notably, this association is present on both basic and complex emotional aspects of social cognition as a function of PSY diagnosis, and only on cognitive-emotional aspects in ESD individuals. Indeed, emotion perception (i.e., the conscious recognition of basic emotions on facial stimuli) has repeatedly been found as impaired in PSY [60, 61], and previous evidence revealed an association between attachment style and both behavioral and brain correlates of emotion recognition [62]. This interaction was significant only in PSY, most likely because this basic emotion ability tends to worsen in the level of impairments along with disease chronicity and symptom severity [63, 64]. Thus, it may be that the lack of interaction on the FEIT score in ESD is due to their more preserved emotion recognition abilities. On the other hand, the presence of a significant group by classification rate interaction in both PSY and ESD on MSCEIT score is consistent with the fact that parenting and attachment-related factors significantly modulate affect regulation at a more complex level since the early stages of psychosis [65, 66]. The ability to manage emotions, indeed, is one of the four emotional intelligence branches and represents the ability of individuals to handle (both at the thought and behavior levels), their own emotions effectively [67]. Consistently, a core concept of attachment theory is that attachment style strongly influences the appraisal of social cues, thus modulating affect regulation from childhood to adulthood [15].

### Limitations

This study has some limitations. First of all, the small sample sizes across cohorts strongly limit the generalizability of our findings, as they may be overfitted [68, 69]. Second, the use of a retrospective perceived maternal care measure and of a self-report attachment questionnaire could reflect false memories or even re-interpretation of the type of care received [70]. However, previous literature has indicated that both measures are stable over time [57–59, 71], suggesting that they may overall constitute reliable measures. Third, the cross-sectional nature of this study does not allow us to give any realistic prognostic insight into psychosis and its early stages based on these findings, or to speculate on whether the experience of a mental illness impacts on the perception of negative childhood experiences, or vice versa. Longitudinal studies are warranted to investigate the prognostic relevance of our model.

### Conclusions

Despite the difficulty in attributing any causality to the association between psychosis and perceptions of negative childhood experiences, our results support the role of perceived negative parenting and attachment insecurity as general vulnerability characteristics in psychosis and its early phases, both because of their good classification power and because of their association with key socio-cognitive psychosis phenotypes. In this view, they improve the understanding of psychosis environmental risk pathways. Furthermore, if externally validated in larger cohorts, they offer intriguing clinical insights about the possibility of promoting positive parenting and attachment security as psychosis resilience factors within early identification and early intervention programs, especially when other more psychosis-specific environmental risk factors (e.g., childhood adverse events, low socioeconomic status, an affected first-degree relative [56]) are present.

## Supplementary Information

**Additional file 1**. Antonucci LA, et al. – Supplementary Information.

## Data Availability

The datasets used and/or analyzed during the current study are available from the corresponding author on reasonable request.

## Abbreviations

BAC: Balanced accuracy
CV: Cross-validation
ESD: Early stages of disease
FEIT: Facial emotion identification task
FHR: Familial high risk
HC: Healthy controls
MSCEIT: Mayer–Salovey–Caruso emotional intelligence test
PBI: Parental bonding instrument
PSY: Individuals diagnosed with Psychosis
SD: Standard deviation
TASIT: The awareness of social inference test
