# Supplementary material for: Machine learning-based ability to classify psychosis and early stages of disease through parenting and attachment-related variables is associated with social cognition
Source: BMC Psychol. 2021 Mar 23;9:47. doi: 10.1186/s40359-021-00552-3 (PMC7989088; doi:10.1186/s40359-021-00552-3)
Supplement: Supplementary file 1 — Additional file 1. Antonucci LA, et al. – Supplementary Information. [file 40359_2021_552_MOESM1_ESM.docx]

**Antonucci LA, et al. – Supplementary Information**

1. **Sample determination: inclusion and exclusion criteria**

A total of 234 individuals, all Caucasians native of the Apulia region, Italy, participated in the study. The discovery sample was composed of 105 individuals, of which 71 HC and 34 patients with psychosis (PSY). Out of the PSY, 20 were diagnosed with schizophrenia (SCZ) and 14 with bipolar disorder with psychotic manifestations (BD). For study inclusion, PSY had to be under stable antipsychotic treatment for at least one month at the moment of enrollment. Diagnoses of SCZ or BD, or exclusion of any psychiatric diagnosis for HC, was assessed with the Structured Clinical Interview for DSM-IV-TR (SCID) (1).

Moreover, 90 individuals were included in the validation clinical sample (Table 1B). Of those, 60 were HC and 30 were individuals at early stages of disease (ESD) compared with the PSY group. Specifically, 9 were labelled as First Episode of Depression (FED), 9 as First Episode of Psychosis (FEP), and 12 as At-Risk Mental State for psychosis (ARMS).

We decided to include FED individuals into the ESD group because of previous evidence highlighting that ARMS individuals very often experience affective symptoms, to the extent that 41% of them have a comorbid depressive disorder (2, 3); based on this finding and on recent early identification views (4), we decided to have an ESD group including ARMS, FEP, and affective states (i.e., FED). We believe that inclusion of this further group of subjects is more likely to provide more realistic and reliable prognostic validation of the model previously built in PSY (see Methods section 2.5).

Individuals were labelled as FED when they met criteria for major depression within the past 3 months, as established by the SCID. FED were excluded when they had a previous episode of DSM-IV-TR major depression prior to the current or recent episode, and when the duration of the current episode exceeded 24 months. FED were excluded when they underwent antipsychotic medication for more than 90 days (cumulative number of days).

Individuals were identified as FEP when they met criteria for DSM-IV-TR affective or non-affective psychotic episode fulfilled within past 3 months, and when the onset of psychosis happened within the past 24 months. FEP were excluded when they underwent antipsychotic medication for more than 90 days (cumulative number of days).

The ARMS state was defined by: (1) cognitive disturbances (COGDIS), as assessed by the Schizophrenia Proneness Instrument (SPI-A, (5)), and/or (2) ultra-high-risk (UHR) criteria for psychosis, according to the Structured Interview for Psychosis-Risk Syndromes criteria (SIPS, (6)). ARMS were excluded when they had an intake of antipsychotic medication for more than 30 cumulative days, and when they had any intake of antipsychotic medication within the past 3 months before study enrollment.

Furthermore, 26 HC and 13 individuals with a Familial High Risk for psychosis (FHR, i.e., with no DSM IV Axis I diagnosis, but with a first-degree relative affected either by SCZ or by BD – Table 1C) were included in the validation familial risk sample. Exclusion of any psychiatric diagnosis for both FHR and HC was assessed with the SCID.

In all individuals, we assessed socio-economic status with the Hollingshead test (7) and intelligence quotient (IQ) using the Wechsler Adult Intelligence Scale-Revised (8). Furthermore, the Italian version of the Wide Reading Achievement Test (9) was employed to have a measure of premorbid IQ for each participant. All individuals had no history of drug or alcohol abuse within the last 6 months, head trauma with loss of consciousness, or any other clinically significant medical condition.

1. **The Parental Bonding Instrument (PBI)**

Perceived parental bonding was measured using the Parental Bonding Instrument (PBI), which is based on the subjects’ memories of their parents during the first 16 years of life (Parker et al. 1980). According to the principles of attachment theory, Parker (1980) posited that nurturing and parenting abilities may influence the quality of the mother-child relationship. Specifically, the perception of a loving and emotionally close parent seems to favor the development of a secure attachment in the child. On the other hand, a perceived cold and intrusive parent can lead to a problematic child development and to subsequent psychological disorders (George et al. 1985).

The PBI is a 25-item self-report questionnaire investigating two main dimensions, “care” and “overprotection”. The “care” dimension reflects perceived parental warmth, affection, and involvement in contrast to coldness, indifference, and rejection. On the other hand, the “overprotection” dimension reflects perceived parental psychological control and intrusion in contrast to the encouragement of autonomy and independence. Subjects were asked to score both their mother’s and father’s attitudes using a 4-point scale.

1. **The Awareness of Social Inference Test (TASIT)**

To investigate theory of mind, we employed The Awareness of Social Inference Test (TASIT, (10); details about the Italian version employed can be found at (11)). The TASIT is composed of seven scales (positive emotions, negative emotions, sincere, simple sarcasm, paradoxical sarcasm, sarcasm enriched, lie), organized into three sections:

- in the section “emotion recognition”, individuals undergo 28 video-vignettes of professional actors enacting ambiguous scripts representing 7 basic emotions (happy, sad, surprised, angry, anxious, revolted, neutral), and at the end of each vignette they are asked to choose the perceived emotion of a given actor indicated in the answer form;
- the second section, social inference (minimal), allows to investigate the understanding of conversational meanings that are determined by paralinguistic cues (e.g., facial expression, tone of voice, gesture, etc.). Here, individuals are required to watch 15 video-vignettes of everyday either sincere or sarcastic or paradoxical conversational exchanges; for each vignette, individuals are asked 4 comprehension questions, respectively testing the understanding of actors’ beliefs, meaning, intentions, and feelings.
- the last section, social inference (enriched), assesses the ability to use contextual knowledge, like visual and verbal information, to derive meaning. Individuals are asked to undergo 16 video-vignettes, each one including a literally untrue comment. To derive meaning from the vignettes, individuals may employ either only paralinguistic features, or also contextual cues (e.g., visual details indicating the state of affairs, or prologues that reveal the speaker’s true thoughts). For each vignette, individuals are asked 4 comprehension questions, respectively testing the understanding of actors’ beliefs, meaning, intentions, and feelings.

Here is an example of a social inference (enriched) vignette:

Speaker 1: “Yes, Cal has finished his dinner!” (but he has not)

Speaker 2: “No, of course you don’t look fat!” (but he does).

This conversational exchange may be read either as a sarcasm meant to amplify the truth, or as a lie meant to minimize the truth). To make the decision, individuals may use visual edits (e.g. is Cal’s dinner plate full or empty), or other prologues (e.g., Speaker 1 telling a third person that Cal has put on weight).

1. **Machine learning strategy**

To allow for unbiased estimation of the model’s generalizability and prevent information leaking between subjects used for training the models and subjects used for validating decisions (12), we built a double cycle, nested cross-validation (CV) framework (4, 13). Nested CV prevents information leaking between individuals used for training and testing the models (12). Indeed, we split the discovery data first into training and test sets on an outer (CV2) cycle, and then we split the resulting training folds again into an inner (CV1) training and test data cycle (14) (13, 15). Therefore, nested CV induces a strict separation between training and test data. Specifically, parameter optimization is performed within the inner (CV1) cycle, and generalization error estimation is performed only in the outer (CV2) cycle. CV2 samples never visited the classification algorithms during the entire training process (12). In other words, all model training steps that use group-level statistical procedures (e.g. 0-1 scaling and regress out of age, gender and socio-economic status effects) occur only in the inner cycle (CV1) training data. This procedure has been performed to prevent any information leakage between those individuals used for model selection and those employed for model testing. The inner cycle test data are used to pick hyperparameter combinations that provide potentially good model generalization capacity. More specifically, hyperparameters optimization involved testing ranges (and not single values) of the *C* (misclassification cost) and *γ* (kernel width) parameters, within a grid defined by the ranges *C* = [0.0156 - 16] and *γ* = [3.0518^-5^ - 8]. C (11 log-spaced values) and γ (10 log-spaced values) parameters (16, 17) are critically important to classification using Support Vector Machine algorithms, as their regularization defines the decision boundary, i.e. the degree of penalty that is applied when a case is misclassified.

Finally, the outer cycle (CV2) validation data served exclusively the purpose of measuring the models’ generalizability to new, unseen data. Both in inner (CV1) and outer (CV2) CV levels, we employed a 10-fold CV cycle. We extended nested CV to repeated nested CV (18) at both inner and outer cross-validations cycles by randomly permuting the participants within their groups (number of permutations = 10) and repeating the CV cycle for each of these permutations. In other words, based on this cross-validation framework, our discovery dataset was randomly split into 10 CV2 folds, one of these folds was then held out, and the rest went into the CV1 training cycle. NeuroMiner then cycled through all of the CV2 folds, and then it repeated this entire process 10 times (i.e., 10 permutations). For each permutation, it shuffled discovery participants before creating the folds to include different participants in each fold.

Our NeuroMiner machine learning pipeline (Table S1) consisted of the following steps:

1. As many machine learning algorithms are sensitive to scale differences between features, we scaled each variable to a 0-1 range to remove these effects from each training sample matrix. Scaling procedures occurred only within the inner CV cycle.
2. Given that age, gender and socio-economic status have been previously reported as associated with attachment security (19, 20), features were further preprocessed by correcting for age, gender and socio-economic status effects. Specifically, we removed the variance associated with age, gender and socio-economic status from the feature scores within each inner CV fold through partial correlations.
3. Features underwent a stepwise forward variable selection process (21) using a linear Support Vector Machine (SVM)(22). We chose to implement SVM with a linear kernel because it has been demonstrated that SVMs with linear kernels are less prone to overfitting compared with SVMs with non-linear kernels (18). The procedure grew a parsimonious combination of features that optimized the average classification performance of the algorithm across the CV1 training and testing data. More specifically, data entered a greedy forward search wrapper (21) which allows identifying the most parsimonious subset of variables within the variable pool, thus providing maximum prognostic performance with the smallest amount of predictive features. More specifically, the wrapper algorithm used an SVM to evaluate the discriminative value of each feature, then extracted the most discriminative feature and reiterated over the remaining variable pool to select the 2nd best performing variable, which was added to the first one. This process was re-iterated until the optimal variable subspace had been identified. We stopped the variable search when the top 50% of the variables had been extracted by the wrapper. Given the small number of features, the wrapper added single features up to the 50% of the total and then tested the model, following the very same procedures reported in our previous publications (17, 23).
4. The trained model was then applied to the outer CV cycle (CV2) by preprocessing the best discriminative variables using the learned scaling and age, gender and socio-economic variables regress-out, and determining each validation individual’s outcome class (HC vs PSY) through majority voting across all ensemble models. In other words, in each variable evaluation step in the CV1, the SVM algorithm modeled linear relationships between features and classification labels (HC vs. PSY). In the linear kernel space, the SVM optimized a hyperplane that maximized separability between most HC-like and most PSY-like individuals (i.e., the Support Vectors). Based on the trained hyperplane, the algorithm then predicted subjects' classification of the inner CV1 cycle by projecting its data into the learned kernel space and measuring their geometric distance to the decision boundary. This resulted in a decision value and a predicted classification label *per* participant.

The wrapper-based feature selection was carried out for each CV1 training and test sample
and then repeated for every combination of the SVM parameters *C* (misclassification cost) and *γ* (kernel width) within a grid defined by the ranges *C* = [0.0156 - 16] and *γ* = [3.0518^-5^ - 8]. Because of our nested cross-validation framework, we created an ensemble of 100 models (n=10 repetition x k=10 folds*)* for each CV2 partition („CV1 ensemble“). Furthermore, due to the 10 repetitions of the CV2 cycle, we could establish a final out-of-training class membership prediction for a given individual by combining all CV1 ensembles into a larger CV2 ensemble, in which the given individual had not served for model training and optimization at the CV1 level. This ensemble generation procedure has been repeatedly described in our previous works (4, 17) and is a feature of the model generation and validation process implemented in NeuroMiner.

To understand the discriminative utility of the input variables within the final ensemble model, we computed for each feature the probability of being selected by the greedy forward search wrapper for classification purposes within the inner CV loop (see Results in main text).

1. **Permutation analysis**

To assign statistical significance to the observed classification performance of our model, we employed permutation (24). We performed 1000 random permutations of the outcome labels. For each permutation, we retrained all linear SVM models in the repeated nested CV experiment using the respective feature subsets obtained from the observed-label analyses. For each permutation, we accumulated the predictions of the random models into a permuted ensemble prediction for each outer cycle subject. Thus, we built a null distribution of out-of-training classification performance based on Balanced Accuracy (BAC). Finally, we calculated the significance of the observed out-of-training BAC as the number of events where the permuted out-of-training BAC was higher or equal to the observed BAC divided by the number of permutations performed. The significance of the model was determined at α=0.05.

**Supplementary Objects**

Table S1: Full settings of the Support Vector Machine analysis.

| **Software** | NeuroMiner, version 1.0 |
| --- | --- |
| **Cross-Validation** | Double-cycle, nested  Both inner and outer cycles: 10 permutations, 10 folds |
| **Preprocessing**  **Steps** | Step 1: 0-1 scaling  Step 2: feature-wise regress out of age, gender and socio-economic status effects |
| **SVM parameter optimization** | *C* = [0.0156 - 16]  *γ* = [3.0518^-5^ - 8]  kernel = linear |
| **Feature engineering procedures** | Wrapper, greedy feature selection  Wrapper direction: forward  Early stopping: 50% |
| **Evaluation parameter** | Balanced Accuracy |

Table S2: correlation analysis between all features which entered the machine learning algorithm, and each socio-cognitive variable of interest used for association analysis.

**References:**

1. First MB GM, Spitzer RL, Williams JBW. *Guide for the structured clinical interview for DSM-IV axis I disorders-research version.*: New York: Biometrics Research (1996).

2. Fusar-Poli P, Salazar de Pablo G, Correll CU, Meyer-Lindenberg A, Millan MJ, Borgwardt S, et al. Prevention of Psychosis: Advances in Detection, Prognosis, and Intervention. *JAMA psychiatry* (2020) 77(7):755-65. Epub 2020/03/12. doi: 10.1001/jamapsychiatry.2019.4779. PubMed PMID: 32159746.

3. Fusar-Poli P, Nelson B, Valmaggia L, Yung AR, McGuire PK. Comorbid depressive and anxiety disorders in 509 individuals with an at-risk mental state: impact on psychopathology and transition to psychosis. *Schizophr Bull* (2014) 40(1):120-31. Epub 2012/11/28. doi: 10.1093/schbul/sbs136. PubMed PMID: 23180756; PubMed Central PMCID: PMCPMC3885287.

4. Koutsouleris N, Kambeitz-Ilankovic L, Ruhrmann S, Rosen M, Ruef A, Dwyer DB, et al. Prediction Models of Functional Outcomes for Individuals in the Clinical High-Risk State for Psychosis or With Recent-Onset Depression: A Multimodal, Multisite Machine Learning Analysis. *JAMA psychiatry* (2018) 75(11):1156-72. doi: 10.1001/jamapsychiatry.2018.2165. PubMed PMID: 30267047; PubMed Central PMCID: PMC6248111.

5. Schultze-Lutter F. RS, Picker H. , Klosterkotter J. Development and evaluation of the schizophrenia proneness instrument, adult version (SPI-A). *Schizophrenia Research* (2006) 86:4-44.

6. Miller TJ, McGlashan TH, Rosen JL, Cadenhead K, Cannon T, Ventura J, et al. Prodromal assessment with the structured interview for prodromal syndromes and the scale of prodromal symptoms: predictive validity, interrater reliability, and training to reliability. *Schizophr Bull* (2003) 29(4):703-15. Epub 2004/03/03. doi: 10.1093/oxfordjournals.schbul.a007040. PubMed PMID: 14989408.

7. Hollingshead AB, Redlich FC. Social class and mental illness: a community study. 1958. *Am J Public Health* (2007) 97(10):1756-7. Epub 2007/09/27. doi: 10.2105/ajph.97.10.1756. PubMed PMID: 17895405; PubMed Central PMCID: PMCPMC1994199.

8. Wechsler D. *Manual for the Wechsler Adult Intelligence Scale—Revised.* : Psychological Corporation, New York. (1981).

9. Colombo LS, G; Brivio, C. Stima del quoziente intellettivo tramite l'applicazione del TIB (Test di Intelligenza Breve). *Giornale Italiano di Psicologia* (2002) (3):613-37.

10. McDonald S, Bornhofen C, Shum D, Long E, Saunders C, Neulinger K. Reliability and validity of The Awareness of Social Inference Test (TASIT): a clinical test of social perception. *Disabil Rehabil* (2006) 28(24):1529-42. Epub 2006/12/21. doi: 10.1080/09638280600646185. PubMed PMID: 17178616.

11. Rocca P, Galderisi S, Rossi A, Bertolino A, Rucci P, Gibertoni D, et al. Social cognition in people with schizophrenia: a cluster-analytic approach. *Psychol Med* (2016) 46(13):2717-29. Epub 2016/09/21. doi: 10.1017/S0033291716001100. PubMed PMID: 27649341.

12. Ruschhaupt M, Huber W, Poustka A, Mansmann U. A compendium to ensure computational reproducibility in high-dimensional classification tasks. *Statistical applications in genetics and molecular biology* (2004) 3:Article37. doi: 10.2202/1544-6115.1078. PubMed PMID: 16646817.

13. Koutsouleris N, Kahn RS, Chekroud AM, Leucht S, Falkai P, Wobrock T, et al. Multisite prediction of 4-week and 52-week treatment outcomes in patients with first-episode psychosis: a machine learning approach. *The lancet Psychiatry* (2016) 3(10):935-46. doi: 10.1016/S2215-0366(16)30171-7. PubMed PMID: 27569526.

14. Varma S, Simon R. Bias in error estimation when using cross-validation for model selection. *BMC bioinformatics* (2006) 7:91. doi: 10.1186/1471-2105-7-91. PubMed PMID: 16504092; PubMed Central PMCID: PMC1397873.

15. Filzmoser P LB, Varmuza K. . Repeated double cross validation. *J Chemometrics* (2009) 23:160-71.

16. Antonucci LA, Penzel N, Pergola G, Kambeitz-Ilankovic L, Dwyer D, Kambeitz J, et al. Multivariate classification of schizophrenia and its familial risk based on load-dependent attentional control brain functional connectivity. *Neuropsychopharmacology* (2020) 45(4):613-21. Epub 2019/10/04. doi: 10.1038/s41386-019-0532-3. PubMed PMID: 31581175; PubMed Central PMCID: PMCPMC7021788.

17. Antonucci LA, Pergola G, Pigoni A, Dwyer D, Kambeitz-Ilankovic L, Penzel N, et al. A Pattern of Cognitive Deficits Stratified for Genetic and Environmental Risk Reliably Classifies Patients With Schizophrenia From Healthy Control Subjects. *Biol Psychiatry* (2020) 87(8):697-707. Epub 2020/01/18. doi: 10.1016/j.biopsych.2019.11.007. PubMed PMID: 31948640.

18. Dwyer DB, Falkai P, Koutsouleris N. Machine Learning Approaches for Clinical Psychology and Psychiatry. *Annual review of clinical psychology* (2018) 14:91-118. doi: 10.1146/annurev-clinpsy-032816-045037. PubMed PMID: 29401044.

19. Del Giudice M. Sex, attachment, and the development of reproductive strategies. *Behav Brain Sci* (2009) 32(1):1-21; discussion -67. Epub 2009/02/13. doi: 10.1017/S0140525X09000016. PubMed PMID: 19210806.

20. van IJzendoorn M.H. B-KM. Invariance of adult attachment across gender, age, culture, and socioeconomic status? *Journal of Social and Personal Relationships* (2010) 27(2):200-8.

21. Saeys Y, Inza I, Larranaga P. A review of feature selection techniques in bioinformatics. *Bioinformatics* (2007) 23(19):2507-17. doi: 10.1093/bioinformatics/btm344. PubMed PMID: 17720704.

22. Noble WS. What is a support vector machine? *Nature biotechnology* (2006) 24(12):1565-7. doi: 10.1038/nbt1206-1565. PubMed PMID: 17160063.

23. Antonucci LA, Taurino A, Laera D, Taurisano P, Losole J, Lutricuso S, et al. An Ensemble of Psychological and Physical Health Indices Discriminates Between Individuals with Chronic Pain and Healthy Controls with High Reliability: A Machine Learning Study. *Pain Ther* (2020) 9(2):601-14. Epub 2020/09/04. doi: 10.1007/s40122-020-00191-3. PubMed PMID: 32880867; PubMed Central PMCID: PMCPMC7648771.

24. Golland P, Fischl B. Permutation tests for classification: towards statistical significance in image-based studies. *Information processing in medical imaging : proceedings of the conference* (2003) 18:330-41. PubMed PMID: 15344469.
